# Neoadjuvant chemotherapy and pathologic response: a retrospective cohort

**DOI:** 10.1590/S1679-45082013000400007

**Published:** 2013

**Authors:** Diocésio Alves Pinto de Andrade, Gustavo Zucca-Matthes, René Aloísio da Costa Vieira, Cristiane Thomaz de Aquino Exel de Andrade, Allini Mafra da Costa, Aurélio Julião de Castro Monteiro, Lissandra Dal Lago, João Soares Nunes

**Affiliations:** 1 Instituto Oncológico de Ribeirão Preto, Ribeirão Preto, SP, Brazil; 2 Hospital de Câncer de Barretos, Barretos, SP, Brazil; 3 Institut Jules Bordet, Brussels, Belgium

**Keywords:** Neoadjuvant therapy, Neoplasm of the breast/chemotherapy, Doxorubicin/administration & dosage, Cyclophosphamide/administration & dosage, Paclitaxel/administration & dosage, Treatment outcome

## Abstract

**Objective:**

To evaluate the complete pathologic response attained by patients diagnosed with locally advanced breast cancer submitted to neoadjuvant chemotherapy based on the doxorubicin/ cyclophosphamide regimen followed by paclitaxel.

**Methods:**

A retrospective cohort of patients with locally advanced breast cancer, admitted to the *Hospital de Câncer de Barretos* between 2006 and 2008 submitted to the doxorubicin/cyclophosphamide protocol followed by paclitaxel (4 cycles of doxorubicin 60mg/m^2^ and cyclophosphamide 600mg/m^2^ every 21 days; 4 cycles of paclitaxel 175mg/m^2^ every 21 days). The following variables were assessed: age, menopause, performance *status*, initial clinical staging, anthropometric data, chemotherapy (dose – duration), toxicity profile, post-treatment staging, surgery, pathologic complete response rate, disease-free survival, and pathological characteristics (type and histological degree, hormonal profile and lymph node involvement). Statistical analysis was performed using a 5% level of significance.

**Results:**

Of the 434 patients evaluated, 136 were excluded due to error in staging or because they had received another type of chemotherapy. Median age was 50 years, all with performance status 0-1. Median initial clinical size of tumor was 65mm and the median final clinical size of the tumor was 22mm. Fifty-one (17.1%) patients experienced a pathologic complete response. Those with a negative hormonal profile or who were triple-negative (negative Her-2 and hormonal profile) experienced a favorable impact on the pathologic complete response.

**Conclusion:**

Neoadjuvant chemotherapy with doxorubicin/ cyclophosphamide followed by paclitaxel provided a pathologic complete response in the population studied in accordance with that observed in the literature. Triple-negative patients had a greater chance of attaining this response.

## RESUMO

**Objetivo:**

Avaliar a taxa de resposta patológica completa atingida pelas pacientes com diagnóstico de câncer de mama localmente avançado submetidas à quimioterapia neoadjuvante baseada no esquema doxorrubicina/ciclofosfamida seguido de paclitaxel.

**Métodos:**

Coorte retrospectiva de pacientes admitidas no Hospital de Câncer de Barretos com câncer de mama localmente avançado entre 2006 e 2008 submetidas ao protocolo de doxorrubicina/ ciclofosfamida seguido de paclitaxel (4 ciclos de doxorrubicina 60mg/m^2^ e ciclofosfamida 600mg/m^2^ a cada 21 dias; 4 ciclos de paclitaxel 175mg/m^2^ a cada 21 dias). As seguintes variáveis foram avaliadas: idade, menopausa, *performance status,* estadiamento clínico inicial, dados antropométricos, quimioterapia (dose – duração), perfil de toxicidade, estadiamento clínico pós-tratamento, cirurgia, resposta patológica completa, sobrevida livre de doença e características anatomopatológicas (tipo e grau histológico, perfil hormonal e comprometimento linfonodal). A análise estatística foi realizada considerando-se o nível de significância de 5%.

**Resultados:**

Das 434 pacientes avaliadas, 136 foram excluídas por erro no estadiamento ou por terem recebido outro tipo de quimioterapia. A mediana de idade foi 50 anos, todas com *performance status* 0-1. A mediana do tamanho clínico inicial do tumor foi 65mm e a mediana do tamanho clínico final do tumor foi 22mm. Apresentaram resposta patológica completa 51 (17,1%) pacientes. Aquelas que apresentavam perfil hormonal negativo ou que eram triplo-negativas (Her-2 e perfil hormonal negativos) tiveram impacto favorável na resposta patológica completa.

**Conclusão:**

Quimioterapia neoadjuvante com doxorrubicina/ciclofosfamida seguidas de paclitaxel ofereceu taxa de resposta patológica completa na população estudada de acordo com a literatura. Pacientes triplo-negativas tiveram maior chance de atingir essa resposta.

## INTRODUCTION

Breast cancer is the second most frequent cancer worldwide and the first among women, excluding non-melanoma skin cancer^(1)^. According to data from the *Instituto Nacional de Câncer* (INCA) for 2012, the estimate was 52,680 new breast cancer cases diagnosed in Brazil, and 15,620 of which would occur in the State of São Paulo, where the incidence rate is 71.7/100 thousand women^(2)^.

The definition of locally advanced breast cancer (LABC) is classically restricted to stage III. This type of patients, along with a change in TNM (Tumour, Node and Metastasis) staging in 2002, which established stage IIIC as locally advanced, and of the inclusion of stage II patients (T3N0 or with T2>3cm) within this context, has made difficult the interpretation of results in neoadjuvant chemotherapy studies (NAC)^(3,4)^.

Although LABC represents 5% of the cases diagnosed by mammography^(5)^, its incidence may be up to 50% among women in developing countries.

Neoadjuvant chemotherapy has some potential advantages: it treats the systemic micrometastatic disease from the beginning; reduces the tumor load; increases the rate of conservative surgery; allows the *in vivo* evaluation of sensitivity to chemotherapy (CT) and a rapid modification of the therapeutic regimen, if necessary^(6)^.

Over the last years, three large prospective and randomized studies attempted to define the role and the best mode of NAC for breast cancer. The NSABP B-18, EORTC 10902, and ECTO compared NAC *versus* adjuvant systemic chemotherapy (ADJ) in patients with breast cancer in the most varied stages of the disease. Results showed a similar overall survival and higher rates of conservative surgery with lower morbidity for the group that had NAC^(4,7,8)^. These data were confirmed by a meta-analysis published in 2005^(9)^.

New studies evaluated the therapeutic response of combination therapy with anthracycline-taxane *versus* anthracycline alone^(10-15)^. Combination CT regimens, whether simultaneous or sequential, may duplicate the pathologic complete response rate (pCR), reaching 30% in a population that includes initial stages^(16,17)^.

The pCR was recently defined as absence of the invading carcinoma on the pathological examination of the breast tissue and axillary lymph nodes^(18)^. Complete response to chemotherapy is associated with longer disease-free survival (DFS) when compared to non-responders^(10,19)^. Between 30 and 50% of complete clinical responses (cCR) had been pathologically confirmed in prior studies^(4,10)^. The value of the predictive response factors within the neoadjuvant scenario is still uncertain, as there are some conflicting results in literature^(20)^.

## OBJECTIVE

The objective of this study was to retrospectively evaluate the pathological complete response achieved by patients with locally advanced breast cancer exposed to neoadjuvant chemotherapy with a sequential regimen based on anthracycline and taxane, and to also correlate this response with clinical factors and biological markers.

## METHODS

A retrospective cohort of patients diagnosed with LABC submitted to at least one cycle of NAC (doxorubicin/ cyclophosphamide protocol – AC – followed by paclitaxel – T) at the *Fundação Pio XII – Hospital de Câncer de Barretos,* between 2006 and 2008. The diagnosis of LABC was defined as all stage III tumors or those that were primarily operable and that were submitted to at least one cycle of NAC from the AC-T protocol.

The clinical charts were retrieved by the Statistics and Medical Chart Storage Service (SAME). Data collection was performed in a standardized manner by means of a previously approved form and by a single trained investigator.

The neoadjuvant standard treatment evaluated was AC-T, which consisted of 4 cycles of AC (doxorubicin 60mg/m^2^ and cyclophosphamide 600mg/m^2^) every 21 days followed by 4 cycles of T (paclitaxel 175mg/m^2^) every 21 days.

The following variables were assessed: age, menopause, performance *status,* initial clinical staging, anthropometric data, CT (dose–duration), toxicity profile, post-treatment staging, surgery, pCR, DFS, and pathological characteristics (type and histological degree, hormonal profile – RH, Her-2, and lymph node involvement).

DFS was defined as the period between the date of surgery and the date of disease relapse (including distant metastases, local and regional recurrence, and contralateral breast cancer) or death, whichever occurred first.

RH and Her-2 were evaluated by immunohistochemistry in the diagnosis of breast cancer. A receptor was considered hormone positive if it was positive for estrogen receptors (ER) and/or progesterone receptors (PR); it was considered Her-2 positive if Her-2 3+; it was considered negative if Her-2 1+ or 2+.

The clinical response of the tumor after the last cycle of NAC was based on the description of the physical examination made with a caliper. We considered as cCR the disappearance of breast lesions and the absence of palpable lymph nodes upon physical examination, and progression of the disease with a >20% increase in the longest diameter of the initial lesions before CT, or the appearance of new lesions during treatment. We defined pCR as the absence of invading tumor in the postoperative specimen, as well as the absence of lymph nodes involved with the tumor.

The NAC was evaluated by means of dose intensity, which is the percentage of the drug offered to the patient relative to the standard protocol dose. Toxicity of the treatment was graded according to the Common Terminology Criteria for Adverse Events (CTCAE), version 3.0.

Statistical analysis was performed using mean, median, and standard deviation (SD) for continuous variables and the χ^2^ test to assess the influence of the hormone profile, Her-2, menopause, age, and histological grade on the pathologic response of the tumor. The level of significance for rejection of the null hypothesis was always equal to or less than 0.05 (5%). For calculations, the Statistical Package for Social Sciences (SPSS) for Windows®, version 17.0 was used.

The study # 112/2007 was approved by the Research Ethics Committee of the *Fundação Pio XII – Hospital do Câncer de Barretos.*

## RESULTS

A total of 434 patients were retrospectively included. One hundred and thirty-six (31.3%) patients were excluded: 66 (48.5%) because they had received other CT regimes, 37 (27.2%) due to metastatic disease upon diagnosis, and 33 (24.3%) because of incomplete clinical/pathological data. Two hundred and ninety-eight patients were analyzed for the endpoints. Clinical and pathological characteristics of the patients are described on table 1.

**Table 1:** Characteristics of the population studied and of chemotherapy doses

Variables		n (%)
Median age (years)		49.4 (21-79)
Menopause		51
BMI		27.0 (17.3-47.8)
Histological type		
	Ductal carcinoma	256 (85.9)
	Lobular carcinoma	27 (9.1)
	Others	9 (3.0)
Histological grade		
	Grade 1	8 (2.7)
	Grade 2	197 (66.1)
	Grade 3	77 (25.8)
Immunohistochemical profile		
	ER/PR positive	197 (66.1)
	Her-2 positive	78 (26.2)
	Triple-negative	73 (24.5)
Doxorubicin dose (mg)		400 (230-600)
Cyclophosphamide dose (mg)		4000 (2,300-4,960)
Paclitaxel dose (mg)		1120 (500-1,960)
Initial tumor (mm)		65 (0-200)
Post-AC tumor (mm)		40 (0-105)
Final tumor (mm)		22 (0-120)
Surgical specimen tumor (mm)		23 (0-145)
Dissected lymph nodes (median)		15 (1-51)
Affected lymph nodes (median)		1 (0-33)

BMI: body mass index; AC: doxorubicin/cyclophosphamide protocol.

Evaluation of the clinical response to chemotherapy is described on table 2. The median of the initial clinical size of the tumor, after four cycles of AC, and after four cycles of T was, respectively, 65, 40, and 22mm. Two hundred and twenty-four (75.2%) patients presented down-staging of the primary tumor.

**Table 2:** Staging variations according to treatment

Staging (TNM)*	Initial (%)	Final (%)	Pathological (%)
0	0	38.4	17.1
I	0	7.9	11.1
IIa	0.3	28.3	17.8
IIb	2	12.5	12.4
IIIa	52.7	5.7	15.8
IIIb	40.9	6.8	12.4
IIIc	4.0	0	13.1
IV	0	0.4	0.3

* Classification of Malignant Tumors, TNM: Tumour, Node and Metastasis

Intensity of the mean dose of doxorubicin was 92%, of cyclophosphamide it was 92%, and of paclitaxel it was 91.3%. Grade 3/4 toxicity occurred in 7.7% of the patients during the AC regime and in 6% during the T regimen. There were 24 cases of neutropenia and 9 cases of neuropathy.

The operation used most often was mastectomy, performed in 237 (79.5%) patients. All patients were submitted to axillary dissection, with a median of 15 (1-51) lymph nodes dissected. The median of interval between the end of the CT and the date of surgery was 39 days (4-687).

In the immunohistochemical profile of the biopsies performed, 197 (66.1%) tumors had a positive hormonal receptor *status* (estrogen and/or progesterone). In 189 (96.9%) cases, patients received adjuvant tamoxifen. Hyperexpression of the Her-2 molecule was found in 71 (23.8%) tumors. Only 26 (36.6%) of these patients received adjuvant therapy with trastuzumab.

The pCR was shown in 51 (17.1%) patients. Another 11 patients presented with a primary pCR tumor, but the lymph nodes were positive. One hundred and twenty-six (42.3%) patients presented no neoplasic involvement in the axilla. Median tumor size in the postoperative specimen was 23mm. Grade 2 histology was found in 60% of tumors, and grade 3, in 27.7%. Lymphovascular infiltration was present in 49.5% of surgical specimens analyzed, and 21.6% of patients presented with skin affected by breast cancer cells.

The median follow-up was 20.7 (4.6 to 45.6) months and median DFS was 16.5 (4.0 to 35.0) months. There were 22 deaths related to tumor recurrence and 235 patients (78.9%) were alive with no evidence of the disease. Of the 63 (21.1%) patients with relapse, 21 were local-regional and 2 in the contralateral breast. The most frequent sites of systemic recurrence were bones (41.3%), lungs (19.0%), central nervous system (15.9%), and liver (14.3%).

A univariate analysis was performed for the following factors: menopause, age (<35 years or ≥35 years), hormonal receptor status, superexpression of Her-2, histological grade (≤2 or 3), and triple-negative profile. Of these, the only one that influenced pCR with statistical significance was the triple-negative profile (Table 3), with a 3.3-fold hazard ratio, favorable to this profile.

**Table 3:** Assessment of pathologic complete response by subgroup

Groups	Total	pCR* n (%)
Triple-negative	73	23 (31.52)
HER-2 -	220	38 (17.3)
HER-2 +	78	13 (16.7)
Hormone receptor -	101	32 (31.7)
Hormone receptor +	197	19 (9.6)

pCR: pathologic complete response.

## DISCUSSION

Despite being a retrospective study, this was the largest cohort of patients submitted to the same NAC treatment for LABC carried out in Brazil^(21,22)^.

Many studies demonstrated that the use of taxanes in a sequential manner in neoadjuvant chemotherapy treatment of breast cancer more than doubles the rate of pCR^(10-12)^. In the study made by Green et al., the control arm was a chemotherapy regimen very similar to the one used in this present study, with a slightly higher dose of paclitaxel (50mg/m^2^ per cycle) and inverting the order of administration of taxane with anthracycline. The pCR found in the American study was 14%. The most significant side effects were febrile neutropenia (19.7%), grade 3 myalgia (29.9%), and grade 3 peripheral neuropathy (14.2%), well above the rates shown in the present study (6 to 7.7%)^(15)^.

Despite the fact that the clinical response has an important subjective component, in this study an increase in the cCR rate was shown when compared to the end of the AC regimen phase and the end of the paclitaxel regime (15.3% → 36%).

A variety of results may be used to assess the influence of NAC on breast cancer, such as overall response, pathological response, clinical response, and radiological response.

The pCR is the most important predictive factor for survival^(23)^. The pCR rate in this study (17.1%) is in accordance with that observed in literature. The percentage of pCR in the prospective phase II and III studies varied between 15% and 30%, with greater emphasis on the study arms that used sequential CT based on weekly docetaxel^(9,10)^ or paclitaxel^(15)^.

In comparison with north american or european trials, the initial tumor size (median of 65mm) was quite a bit larger in this study, a fact influenced by the socio-economic condition of the population and by the precarious public healthcare system found in some areas of our country, such as the North and Northeast. Another fact that demonstrates the delay in diagnosis is that only 27 patients (8.6%) presented with clinically negative axilla.

Due to a short median follow-up of 20.6 months, evaluation of overall and disease-free survival was hindered. However, studies with NAC are an ideal scenario for determining prognostic and predictive factors of treatment response, since biopsies may be performed before starting CT and compared to the surgically excised tumor. Some examples of pharmacogenomic markers that may be correlated to the CT response, the Her-2 *status*, the hormone *status*, and p53 and Ki67 levels, among others^(24)^. The triple-negative subgroup showed a pCR rate of 31.5%, and was considered a predictive factor of response to NAC treatment in this population.

The future of NAC resides in the capacity to predict the response to certain chemotherapy agents so that treatment can be adapted for maximal benefit to the patient, producing better long-term results.

## CONCLUSION

Corroborating data found in literature of prospective studies on neoadjuvant breast cancer treatment, the subgroup that showed the highest percentage of pCR, related to NAC, with a sequential regimen based on anthracycline and taxane, was triple-negative, and this group was a predictor of response to the treatment given. Due to the short mean follow-up time, it was not possible to determine the prognosis of the subgroups evaluated. This cohort demonstrated characteristics of a portion of the Brazilian population in response to a worldwide standard chemotherapy treatment regime.
